# Crystal structure of the enol form of mesotrione: a benzoyl­cyclo­hexa­nedione herbicide

**DOI:** 10.1107/S2056989015012803

**Published:** 2015-07-08

**Authors:** Gihaeng Kang, Jineun Kim, Hyunjin Park, Tae Ho Kim

**Affiliations:** a Department of Chemistry and Research Institute of Natural Sciences, Gyeongsang National University, Jinju 660-701, Republic of Korea

**Keywords:** crystal structure, tautomerization, enol form, intra­molecular O—H⋯O hydrogen bond.

## Abstract

The title compound [systematic name: 3-hy­droxy-2-(4-methyl­sulfonyl-2-nitro­benzo­yl)cyclo­hex-2-enone], C_14_H_13_NO_7_S, is the enol form of a benzoyl­cyclo­hexa­nedione herbicide. As a result of this tautomerization, there is intra­molecular O—H⋯O hydrogen bond enclosing an *S*(6) ring motif. The cyclo­hexene ring has an envelope conformation, with the central CH_2_ C atom as the flap. Its mean plane is inclined to the benzene ring by 87.46 (8)°. In the crystal, mol­ecules are linked by a series of C—H⋯O hydrogen bonds, forming a three-dimensional framework.

## Related literature   

For information on the herbicidal properties of the title compound, see: Mitchell *et al.* (2001 ▸). For related crystal structures, see: Eftekhari-Sis *et al.* (2012 ▸); Liu & Tang (2012 ▸).
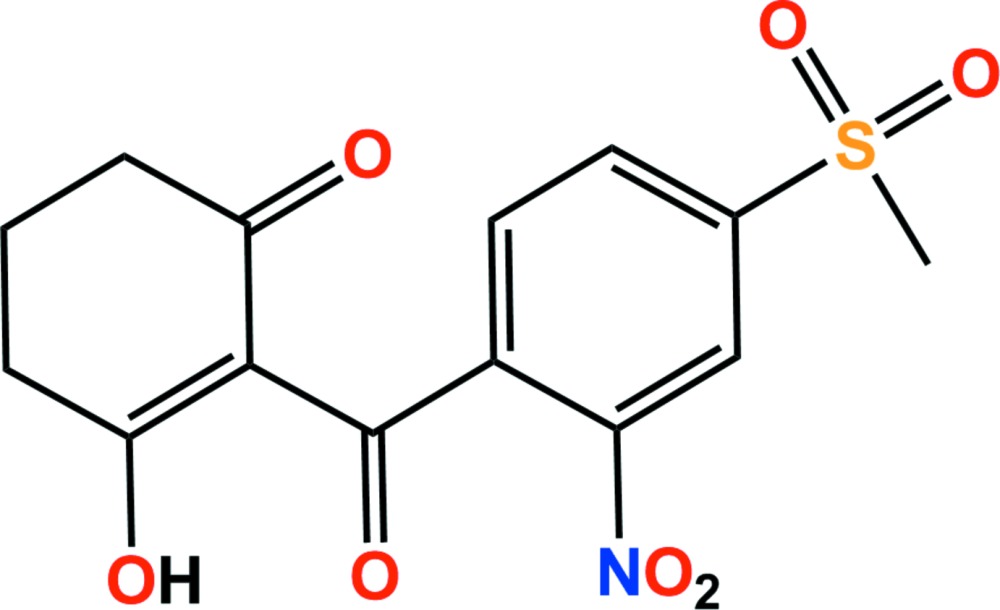


## Experimental   

### Crystal data  

C_14_H_13_NO_7_S
*M* *_r_* = 339.31
Monoclinic, 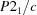

*a* = 10.4208 (2) Å
*b* = 11.2525 (3) Å
*c* = 12.3550 (3) Å
β = 95.370 (1)°
*V* = 1442.39 (6) Å^3^
*Z* = 4
Mo *K*α radiation
μ = 0.26 mm^−1^
*T* = 173 K
0.43 × 0.30 × 0.20 mm

### Data collection  

Bruker APEXII CCD diffractometer
Absorption correction: multi-scan (*SADABS*; Bruker, 2009 ▸) *T*_min_ = 0.895, *T*_max_ = 0.949
12093 measured reflections
2828 independent reflections
2572 reflections with *I* > 2σ(*I*)
*R*_int_ = 0.025

### Refinement  

*R*[*F* ^2^ > 2σ(*F* ^2^)] = 0.036
*wR*(*F* ^2^) = 0.097
*S* = 1.04
2828 reflections
213 parameters
H atoms treated by a mixture of independent and constrained refinement
Δρ_max_ = 0.48 e Å^−3^
Δρ_min_ = −0.40 e Å^−3^

### 

Data collection: *APEX2* (Bruker, 2009 ▸); cell refinement: *SAINT* (Bruker, 2009 ▸); data reduction: *SAINT*; program(s) used to solve structure: *SHELXS97* (Sheldrick 2008 ▸); program(s) used to refine structure: *SHELXL2013* (Sheldrick, 2015 ▸); molecular graphics: *DIAMOND* (Brandenburg, 2010 ▸); software used to prepare material for publication: *SHELXTL* (Sheldrick 2008 ▸).

## Supplementary Material

Crystal structure: contains datablock(s) global, I. DOI: 10.1107/S2056989015012803/su5166sup1.cif

Structure factors: contains datablock(s) I. DOI: 10.1107/S2056989015012803/su5166Isup2.hkl

Supporting information file. DOI: 10.1107/S2056989015012803/su5166Isup3.cml

**Fig. 1.**
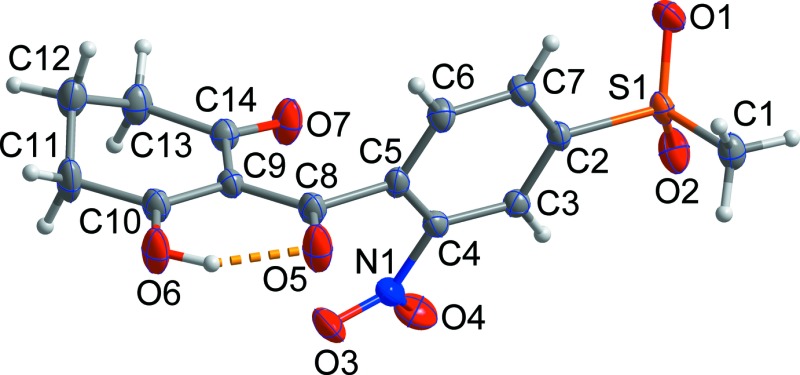
The molecular structure of the title compound, with atom labelling. Displacement ellipsoids are drawn at the 50% probability level. The intramolecular O—H···O hydrogen bond is shown as a dashed line (see Table 1 for details).

**Fig. 2.**
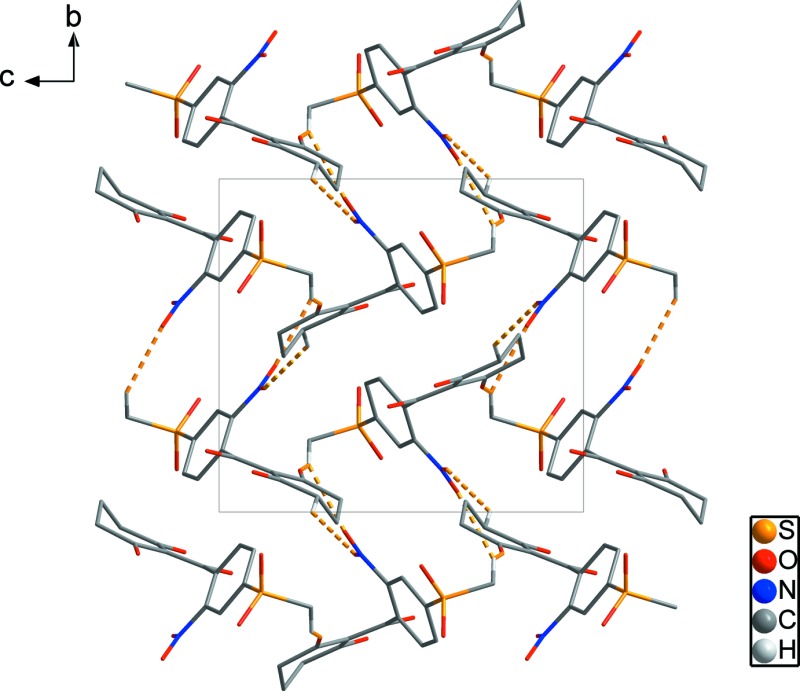
Crystal packing of the title compound viewed along the a axis. The intermolecular C—H···O hydrogen bonds are shown as dashed lines (see Table 1 for details).

. DOI: 10.1107/S2056989015012803/su5166fig1.tif
The mol­ecular structure of the title compound, with atom labelling. Displacement ellipsoids are drawn at the 50% probability level. The intra­molecular O—H⋯O hydrogen bond is shown as a dashed line (see Table 1 for details).

a . DOI: 10.1107/S2056989015012803/su5166fig2.tif
Crystal packing of the title compound viewed along the *a* axis. The inter­molecular C—H⋯O hydrogen bonds are shown as dashed lines (see Table 1 for details).

CCDC reference: 1410192

Additional supporting information:  crystallographic information; 3D view; checkCIF report

## Figures and Tables

**Table 1:** Hydrogen-bond geometry (, )

*D*H*A*	*D*H	H*A*	*D* *A*	*D*H*A*
O6H6*O*O5	0.91(3)	1.71(3)	2.524(2)	148(2)
C1H1*B*O4^i^	0.98	2.58	3.393(2)	140
C1H1*B*O7^ii^	0.98	2.58	3.265(2)	127
C11H11*A*O3^iii^	0.99	2.40	3.135(2)	131

Symmetry codes: (i) 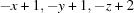; (ii) 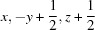; (iii) 
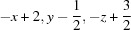
.

## supplementary crystallographic information

### S1. Comment

Mesotrione, [keto form systematic name: 2-(4-mesyl-2-nitrobenzoyl)cyclohexane-1,3-dione], is a benzoylcyclohexanedione herbicide and it has been developed for the selective pre- and post-emergence control of a wide range of broad-leaved and grass weeds in maize (Mitchell *et al.*, 2001). However, until now its crystal structure has not been reported.

The title compound crystallized in the enol form (Fig. 1 and Table 1), with an intramolecular O6—H6O···O5 hydrogen bond embedded in an *S*(6) ring. The cyclohexene ring has an envelope conformation with the central CH_2_ C-atom, C12, as the flap. Its mean plane is inclined to the benzene ring by 87.46 (8) °.

All bond lengths and bond angles are normal and comparable to those observed in the crystal structures of similar compounds (Eftekhari-Sis *et al.*, 2012; Liu *et al.*, 2012).

In the crystal, molecules are linked by a series of C—H···O hydrogen bonds forming a three-dimensional framework (Fig. 2 and Table 1).

### S2. Experimental

The title compound was purchased from the Dr. Ehrenstorfer GmbH Company. Slow evaporation of a solution in CH_3_CN gave single crystals suitable for X-ray analysis.

### S3. Refinement

The O-bound H atom was located in a difference Fourier map and freely refined [O—H = 0.91 (3) Å]. The C-bound H atoms were positioned geometrically and refined using a riding model: C-H = 0.95 - 0.99 \%A with *U*_iso_(H) = 1.5*U*_eq_(C) for methyl H atoms and

1.2*U*_eq_(C) for other H atoms.

### Crystal data

**Table:**

C_14_H_13_NO_7_S	*F*(000) = 704
*M**_r_* = 339.31	*D*_x_ = 1.563 Mg m^−^^3^
Monoclinic, *P*2_1_/*c*	Mo *K*α radiation, λ = 0.71073 Å
*a* = 10.4208 (2) Å	Cell parameters from 7168 reflections
*b* = 11.2525 (3) Å	θ = 2.5–27.5°
*c* = 12.3550 (3) Å	µ = 0.26 mm^−^^1^
β = 95.370 (1)°	*T* = 173 K
*V* = 1442.39 (6) Å^3^	Block, colourless
*Z* = 4	0.43 × 0.30 × 0.20 mm

### Data collection

**Table:**

Bruker APEXII CCD diffractometer	2572 reflections with *I* > 2σ(*I*)
φ and ω scans	*R*_int_ = 0.025
Absorption correction: multi-scan (*SADABS*; Bruker, 2009)	θ_max_ = 26.0°, θ_min_ = 2.0°
*T*_min_ = 0.895, *T*_max_ = 0.949	*h* = −12→12
12093 measured reflections	*k* = −13→13
2828 independent reflections	*l* = −15→15

### Refinement

**Table:**

Refinement on *F*^2^	Primary atom site location: structure-invariant direct methods
Least-squares matrix: full	Secondary atom site location: difference Fourier map
*R*[*F*^2^ > 2σ(*F*^2^)] = 0.036	Hydrogen site location: mixed
*wR*(*F*^2^) = 0.097	H atoms treated by a mixture of independent and constrained refinement
*S* = 1.04	*w* = 1/[σ^2^(*F*_o_^2^) + (0.0538*P*)^2^ + 0.7458*P*] where *P* = (*F*_o_^2^ + 2*F*_c_^2^)/3
2828 reflections	(Δ/σ)_max_ < 0.001
213 parameters	Δρ_max_ = 0.48 e Å^−^^3^
0 restraints	Δρ_min_ = −0.40 e Å^−^^3^

### Special details

**Table:**

Geometry. All e.s.d.'s (except the e.s.d. in the dihedral angle between two l.s. planes) are estimated using the full covariance matrix. The cell e.s.d.'s are taken into account individually in the estimation of e.s.d.'s in distances, angles and torsion angles; correlations between e.s.d.'s in cell parameters are only used when they are defined by crystal symmetry. An approximate (isotropic) treatment of cell e.s.d.'s is used for estimating e.s.d.'s involving l.s. planes.

### Fractional atomic coordinates and isotropic or equivalent isotropic displacement parameters (Å^2^)

**Table:**

	*x*	*y*	*z*	*U*_iso_*/*U*_eq_	
S1	0.31056 (3)	0.24106 (4)	1.11672 (3)	0.02141 (13)	
O1	0.24559 (11)	0.12877 (11)	1.11593 (11)	0.0333 (3)	
O2	0.24978 (11)	0.33729 (12)	1.05544 (10)	0.0341 (3)	
O3	0.78663 (13)	0.37212 (12)	0.87472 (13)	0.0461 (4)	
O4	0.59220 (13)	0.44028 (12)	0.84539 (11)	0.0406 (3)	
O5	0.92622 (11)	0.18323 (14)	1.03272 (11)	0.0420 (4)	
O6	1.08825 (11)	0.11994 (14)	0.90444 (11)	0.0387 (3)	
H6O	1.057 (3)	0.151 (2)	0.965 (2)	0.062 (8)*	
O7	0.64741 (11)	0.12478 (13)	0.77772 (11)	0.0367 (3)	
N1	0.67227 (14)	0.37175 (13)	0.88940 (12)	0.0280 (3)	
C1	0.34825 (16)	0.28528 (17)	1.25212 (13)	0.0279 (4)	
H1A	0.2685	0.2995	1.2863	0.042*	
H1B	0.3993	0.3585	1.2540	0.042*	
H1C	0.3981	0.2225	1.2918	0.042*	
C2	0.46359 (14)	0.21719 (14)	1.06875 (12)	0.0195 (3)	
C3	0.51015 (14)	0.30302 (14)	1.00287 (12)	0.0206 (3)	
H3	0.4624	0.3733	0.9847	0.025*	
C4	0.62836 (15)	0.28346 (14)	0.96419 (12)	0.0213 (3)	
C5	0.70348 (14)	0.18398 (15)	0.99181 (13)	0.0224 (3)	
C6	0.65419 (15)	0.09958 (15)	1.05868 (13)	0.0247 (3)	
H6	0.7035	0.0309	1.0792	0.030*	
C7	0.53315 (15)	0.11459 (14)	1.09596 (13)	0.0234 (3)	
H7	0.4986	0.0552	1.1396	0.028*	
C8	0.83866 (15)	0.16470 (15)	0.95976 (14)	0.0258 (4)	
C9	0.86333 (14)	0.11938 (14)	0.85403 (13)	0.0221 (3)	
C10	0.98924 (15)	0.09727 (16)	0.83365 (14)	0.0268 (4)	
C11	1.02509 (16)	0.0464 (2)	0.72964 (16)	0.0378 (5)	
H11A	1.1048	−0.0012	0.7437	0.045*	
H11B	1.0428	0.1117	0.6795	0.045*	
C12	0.91933 (17)	−0.03099 (19)	0.67708 (16)	0.0368 (4)	
H12A	0.9099	−0.1027	0.7220	0.044*	
H12B	0.9416	−0.0569	0.6046	0.044*	
C13	0.79406 (16)	0.03715 (17)	0.66546 (14)	0.0312 (4)	
H13A	0.7239	−0.0180	0.6393	0.037*	
H13B	0.7995	0.0992	0.6093	0.037*	
C14	0.75890 (15)	0.09568 (15)	0.76871 (13)	0.0238 (3)	

### Atomic displacement parameters (Å^2^)

**Table:**

	*U*^11^	*U*^22^	*U*^33^	*U*^12^	*U*^13^	*U*^23^
S1	0.0139 (2)	0.0295 (2)	0.0211 (2)	0.00081 (14)	0.00345 (15)	0.00020 (15)
O1	0.0245 (6)	0.0388 (7)	0.0376 (7)	−0.0102 (5)	0.0082 (5)	−0.0049 (6)
O2	0.0242 (6)	0.0461 (8)	0.0324 (7)	0.0129 (5)	0.0046 (5)	0.0085 (6)
O3	0.0384 (8)	0.0357 (8)	0.0693 (10)	−0.0057 (6)	0.0325 (7)	0.0051 (7)
O4	0.0450 (8)	0.0374 (8)	0.0395 (8)	−0.0054 (6)	0.0042 (6)	0.0145 (6)
O5	0.0205 (6)	0.0733 (10)	0.0320 (7)	−0.0026 (6)	0.0022 (5)	−0.0197 (7)
O6	0.0158 (6)	0.0656 (10)	0.0350 (7)	−0.0005 (6)	0.0033 (5)	−0.0182 (7)
O7	0.0193 (6)	0.0557 (9)	0.0344 (7)	0.0087 (5)	−0.0007 (5)	−0.0121 (6)
N1	0.0321 (8)	0.0249 (7)	0.0286 (8)	−0.0075 (6)	0.0116 (6)	−0.0025 (6)
C1	0.0227 (8)	0.0394 (10)	0.0222 (8)	0.0006 (7)	0.0043 (6)	−0.0043 (7)
C2	0.0155 (7)	0.0250 (8)	0.0185 (7)	−0.0002 (6)	0.0035 (6)	−0.0027 (6)
C3	0.0196 (7)	0.0219 (8)	0.0203 (7)	0.0000 (6)	0.0022 (6)	−0.0026 (6)
C4	0.0220 (8)	0.0236 (8)	0.0190 (7)	−0.0050 (6)	0.0054 (6)	−0.0028 (6)
C5	0.0183 (7)	0.0282 (9)	0.0212 (8)	−0.0014 (6)	0.0039 (6)	−0.0074 (6)
C6	0.0219 (7)	0.0267 (8)	0.0259 (8)	0.0047 (6)	0.0040 (6)	0.0005 (7)
C7	0.0226 (8)	0.0252 (8)	0.0228 (8)	0.0001 (6)	0.0046 (6)	0.0027 (6)
C8	0.0183 (7)	0.0318 (9)	0.0276 (8)	−0.0019 (6)	0.0042 (6)	−0.0052 (7)
C9	0.0181 (7)	0.0249 (8)	0.0240 (8)	−0.0010 (6)	0.0051 (6)	−0.0029 (6)
C10	0.0185 (7)	0.0340 (9)	0.0283 (8)	−0.0013 (7)	0.0044 (6)	−0.0047 (7)
C11	0.0218 (8)	0.0590 (13)	0.0341 (10)	0.0007 (8)	0.0103 (7)	−0.0155 (9)
C12	0.0304 (9)	0.0471 (11)	0.0338 (10)	0.0009 (8)	0.0082 (8)	−0.0131 (8)
C13	0.0269 (8)	0.0437 (10)	0.0227 (8)	0.0024 (7)	0.0006 (6)	−0.0060 (7)
C14	0.0212 (8)	0.0268 (8)	0.0234 (8)	0.0011 (6)	0.0026 (6)	−0.0005 (6)

### Geometric parameters (Å, º)

**Table:**

S1—O1	1.4331 (13)	C5—C6	1.389 (2)
S1—O2	1.4343 (12)	C5—C8	1.514 (2)
S1—C1	1.7543 (17)	C6—C7	1.393 (2)
S1—C2	1.7730 (15)	C6—H6	0.9500
O3—N1	1.2222 (19)	C7—H7	0.9500
O4—N1	1.225 (2)	C8—C9	1.448 (2)
O5—C8	1.239 (2)	C9—C10	1.382 (2)
O6—C10	1.314 (2)	C9—C14	1.467 (2)
O6—H6O	0.91 (3)	C10—C11	1.486 (2)
O7—C14	1.222 (2)	C11—C12	1.504 (3)
N1—C4	1.459 (2)	C11—H11A	0.9900
C1—H1A	0.9800	C11—H11B	0.9900
C1—H1B	0.9800	C12—C13	1.509 (2)
C1—H1C	0.9800	C12—H12A	0.9900
C2—C3	1.380 (2)	C12—H12B	0.9900
C2—C7	1.388 (2)	C13—C14	1.511 (2)
C3—C4	1.380 (2)	C13—H13A	0.9900
C3—H3	0.9500	C13—H13B	0.9900
C4—C5	1.390 (2)		
			
O1—S1—O2	118.50 (8)	C2—C7—H7	120.4
O1—S1—C1	108.66 (8)	C6—C7—H7	120.4
O2—S1—C1	109.73 (8)	O5—C8—C9	122.34 (14)
O1—S1—C2	107.66 (7)	O5—C8—C5	115.18 (14)
O2—S1—C2	107.73 (7)	C9—C8—C5	122.32 (13)
C1—S1—C2	103.51 (7)	C10—C9—C8	118.67 (14)
C10—O6—H6O	107.8 (17)	C10—C9—C14	119.31 (14)
O3—N1—O4	124.40 (15)	C8—C9—C14	122.01 (14)
O3—N1—C4	117.66 (15)	O6—C10—C9	122.94 (15)
O4—N1—C4	117.94 (14)	O6—C10—C11	113.90 (14)
S1—C1—H1A	109.5	C9—C10—C11	123.15 (15)
S1—C1—H1B	109.5	C10—C11—C12	111.30 (14)
H1A—C1—H1B	109.5	C10—C11—H11A	109.4
S1—C1—H1C	109.5	C12—C11—H11A	109.4
H1A—C1—H1C	109.5	C10—C11—H11B	109.4
H1B—C1—H1C	109.5	C12—C11—H11B	109.4
C3—C2—C7	121.38 (14)	H11A—C11—H11B	108.0
C3—C2—S1	117.90 (12)	C11—C12—C13	109.84 (16)
C7—C2—S1	120.72 (12)	C11—C12—H12A	109.7
C2—C3—C4	117.84 (14)	C13—C12—H12A	109.7
C2—C3—H3	121.1	C11—C12—H12B	109.7
C4—C3—H3	121.1	C13—C12—H12B	109.7
C3—C4—C5	122.95 (14)	H12A—C12—H12B	108.2
C3—C4—N1	116.98 (14)	C12—C13—C14	114.66 (14)
C5—C4—N1	120.06 (14)	C12—C13—H13A	108.6
C6—C5—C4	117.75 (14)	C14—C13—H13A	108.6
C6—C5—C8	117.56 (14)	C12—C13—H13B	108.6
C4—C5—C8	124.58 (15)	C14—C13—H13B	108.6
C5—C6—C7	120.71 (15)	H13A—C13—H13B	107.6
C5—C6—H6	119.6	O7—C14—C9	122.24 (15)
C7—C6—H6	119.6	O7—C14—C13	120.09 (14)
C2—C7—C6	119.30 (15)	C9—C14—C13	117.63 (14)
			
O1—S1—C2—C3	141.94 (12)	C6—C5—C8—O5	−73.6 (2)
O2—S1—C2—C3	13.08 (15)	C4—C5—C8—O5	102.5 (2)
C1—S1—C2—C3	−103.11 (13)	C6—C5—C8—C9	101.98 (19)
O1—S1—C2—C7	−37.36 (15)	C4—C5—C8—C9	−81.9 (2)
O2—S1—C2—C7	−166.22 (13)	O5—C8—C9—C10	−0.7 (3)
C1—S1—C2—C7	77.59 (15)	C5—C8—C9—C10	−175.97 (15)
C7—C2—C3—C4	0.3 (2)	O5—C8—C9—C14	178.90 (17)
S1—C2—C3—C4	−179.00 (11)	C5—C8—C9—C14	3.6 (3)
C2—C3—C4—C5	−2.4 (2)	C8—C9—C10—O6	−2.8 (3)
C2—C3—C4—N1	176.38 (13)	C14—C9—C10—O6	177.62 (16)
O3—N1—C4—C3	162.96 (15)	C8—C9—C10—C11	177.56 (17)
O4—N1—C4—C3	−17.2 (2)	C14—C9—C10—C11	−2.0 (3)
O3—N1—C4—C5	−18.3 (2)	O6—C10—C11—C12	151.87 (17)
O4—N1—C4—C5	161.57 (15)	C9—C10—C11—C12	−28.4 (3)
C3—C4—C5—C6	2.0 (2)	C10—C11—C12—C13	53.4 (2)
N1—C4—C5—C6	−176.65 (14)	C11—C12—C13—C14	−51.4 (2)
C3—C4—C5—C8	−174.05 (14)	C10—C9—C14—O7	−172.27 (17)
N1—C4—C5—C8	7.3 (2)	C8—C9—C14—O7	8.1 (3)
C4—C5—C6—C7	0.3 (2)	C10—C9—C14—C13	5.5 (2)
C8—C5—C6—C7	176.70 (14)	C8—C9—C14—C13	−174.07 (16)
C3—C2—C7—C6	2.0 (2)	C12—C13—C14—O7	−160.12 (17)
S1—C2—C7—C6	−178.77 (12)	C12—C13—C14—C9	22.0 (2)
C5—C6—C7—C2	−2.3 (2)		

### Hydrogen-bond geometry (Å, º)

**Table:**

*D*—H···*A*	*D*—H	H···*A*	*D*···*A*	*D*—H···*A*
O6—H6*O*···O5	0.91 (3)	1.71 (3)	2.524 (2)	148 (2)
C1—H1*B*···O4^i^	0.98	2.58	3.393 (2)	140
C1—H1*B*···O7^ii^	0.98	2.58	3.265 (2)	127
C11—H11*A*···O3^iii^	0.99	2.40	3.135 (2)	131

Symmetry codes: (i) −*x*+1, −*y*+1, −*z*+2; (ii) *x*, −*y*+1/2, *z*+1/2; (iii) −*x*+2, *y*−1/2, −*z*+3/2.
