# Supplementary figures and images for: Crystal structure of the enol form of mesotrione: a benzoyl­cyclo­hexa­nedione herbicide
Source: Acta Crystallogr E Crystallogr Commun. 2015 Jul 8;71(Pt 8):o548–9. doi: 10.1107/S2056989015012803 (PMC4571390; doi:10.1107/S2056989015012803)

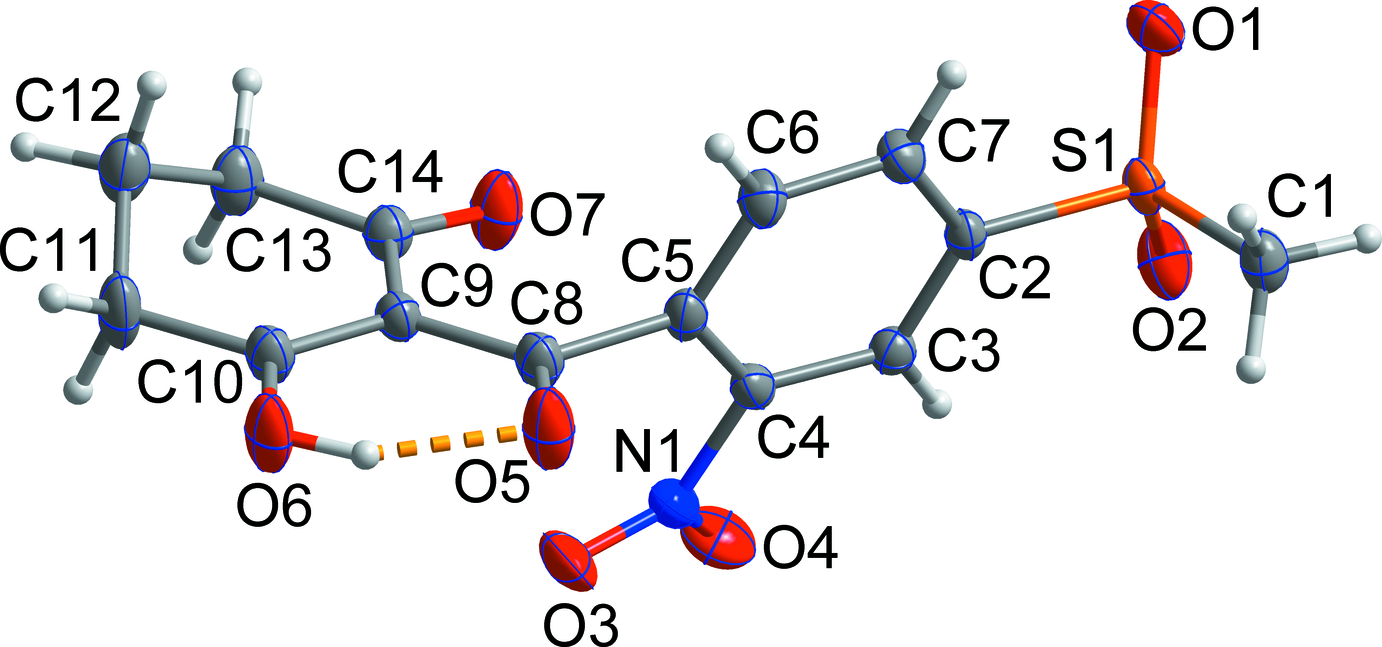

Supplement: Supplementary file 4 [file e-71-0o548-fig1.tif]

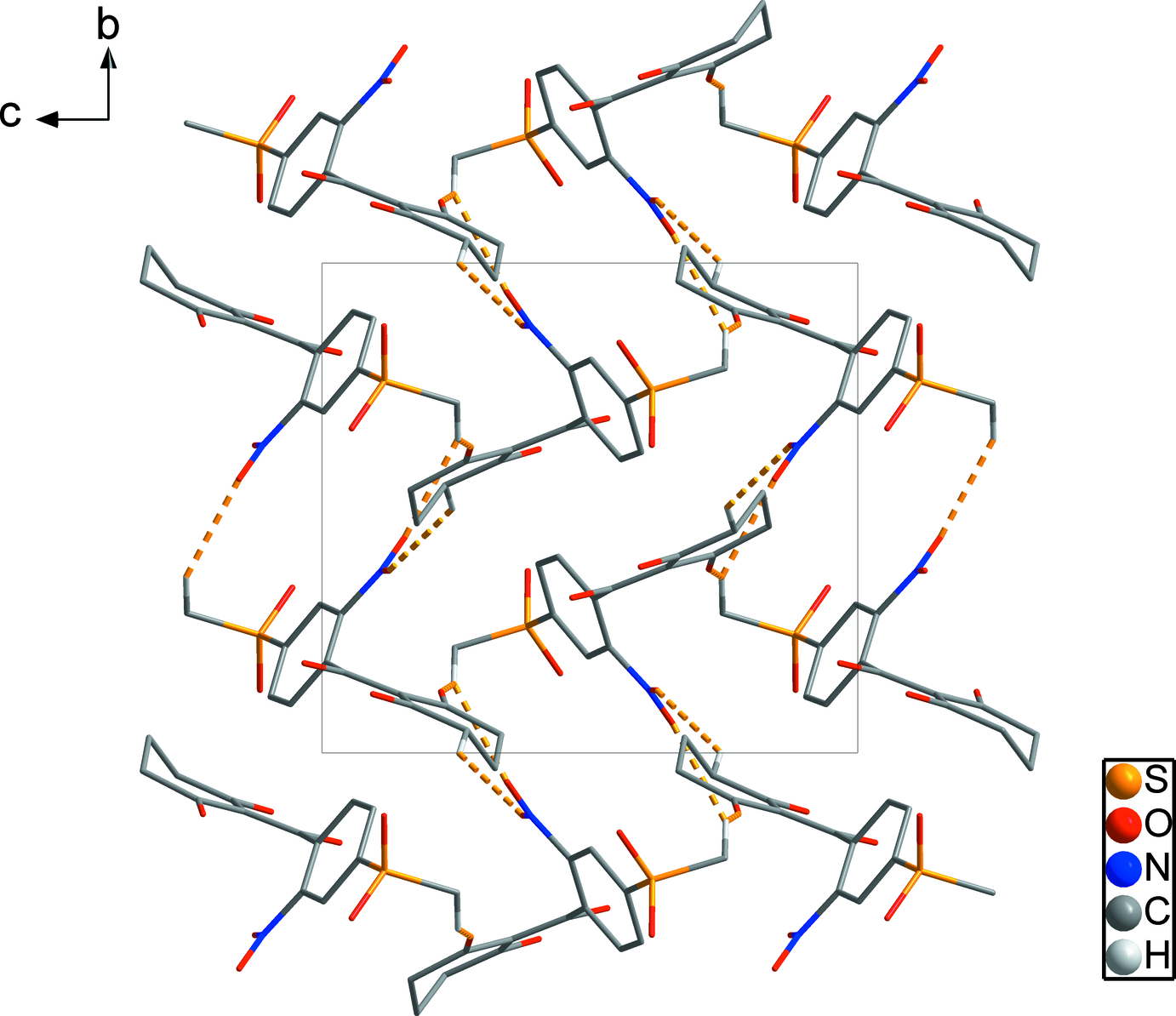

Supplement: Supplementary file 5 [file e-71-0o548-fig2.tif]
